# REJECTION OF CARE AND AGGRESSION AMONG OLDER VETERANS WITH DEMENTIA WITH AND WITHOUT POSTTRAUMATIC STRESS DISORDER

**DOI:** 10.1093/geroni/igac059.1825

**Published:** 2022-12-20

**Authors:** Bada Kang, Wei Pan, Michele Karel, Kirsten Corazzini, Eleanor McConnell

**Affiliations:** Yonsei University, Seoul, Seoul-t'ukpyolsi, Republic of Korea; Duke University, Durham, North Carolina, United States; VA Central Office, Washington, Washington, United States; University of New Hampshire, Durham, New Hampshire, United States; Duke University, Durham, North Carolina, United States

## Abstract

Veterans with co-occurring dementia and posttraumatic stress disorder (PTSD) living in residential long-term care encounter a range of physical and social stimuli, which may trigger trauma-related distress that can be exacerbated and manifested with care rejection and aggression. Yet, it is largely unknown how PTSD influences manifestation of care rejection and aggression in older veterans with dementia. Guided by the need-driven dementia-compromised model, this study examined the moderation effect of PTSD on pathways from background factors, and interpersonal triggers to care rejection and aggression among veterans with dementia with and without co-occurring PTSD. In this secondary analysis study, a multi-group structural equation modeling was conducted using program evaluation data of 315 veterans with dementia from the STAR-VA behavioral intervention implemented in 76 Veterans Health Administration-operated nursing homes. Although no moderation effect of PTSD on the overall model was found, findings revealed distinct patterns of relationships among background factors, interpersonal triggers, and care rejection and aggression between veterans with dementia with and without PTSD. The magnitude of the direct effects of interpersonal triggers on care rejection was greater in veterans with PTSD. Findings on the indirect effect of depression via interpersonal triggers on care rejection and direct effect of functional status on aggression only in veterans with PTSD implies that different mechanisms may underlie distressed behavior depending upon whether or not a veteran has PTSD. This study also underscores the importance of an enhanced focus on trauma-informed care, and individualized multi-component symptom management approach for veterans with dementia and PTSD.

